# Correction: Predictive markers for the early prognosis of dengue severity: A systematic review and meta-analysis

**DOI:** 10.1371/journal.pntd.0010164

**Published:** 2022-01-27

**Authors:** Tran Quang Thach, Heba Gamal Eisa, AlMotsim Ben Hmeda, Hazem Faraj, Tieu Minh Thuan, Manal Mahmoud Abdelrahman, Mario Gerges Awadallah, Nam Xuan Ha, Michael Noeske, Jeza Muhamad Abdul Aziz, Nguyen Hai Nam, Mohamed El Nile, Shyam Prakash Dumre, Nguyen Tien Huy, Kenji Hirayama

There are two errors in the affiliations:

The affiliation for Tran Quang Thach should be: Department of Immunogenetics, Institute of Tropical Medicine (NEKKEN), Nagasaki University, Nagasaki, Japan and Graduate School of Biomedical Sciences, Nagasaki University, Nagasaki, Japan.

The affiliation for Kenji Hirayama should be: Graduate School of Biomedical Sciences, Nagasaki University, Nagasaki, Japan; Department of Immunogenetics, Institute of Tropical Medicine (NEKKEN), Nagasaki University, Nagasaki, Japan; and School of Tropical Medicine and Global Health, Nagasaki University, Nagasaki, Japan.
