# Supplementary figures and images for: Predictive markers for the early prognosis of dengue severity: A systematic review and meta-analysis
Source: PLoS Negl Trop Dis. 2021 Oct 5;15(10):e0009808. doi: 10.1371/journal.pntd.0009808 (PMC8519480; doi:10.1371/journal.pntd.0009808)

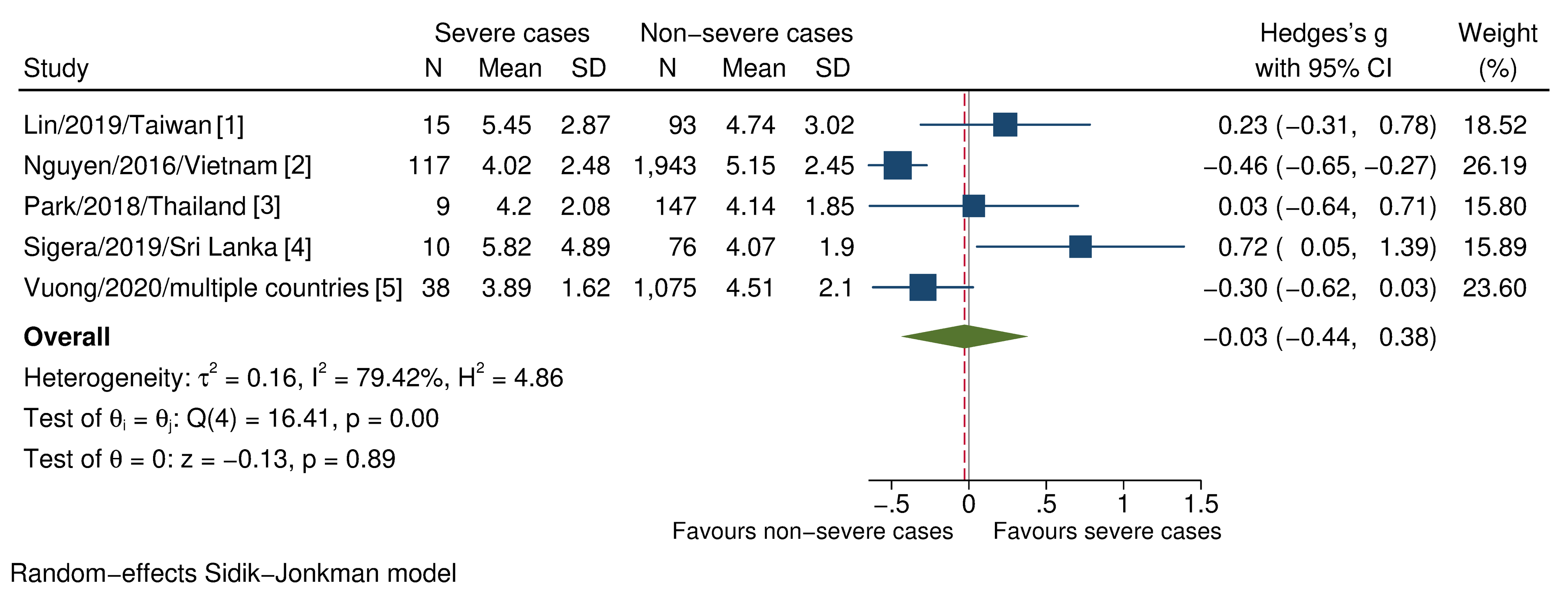

Supplement: S1 Fig — The red dashed line represented the overall effect size. (TIF) [file pntd.0009808.s001.tif]

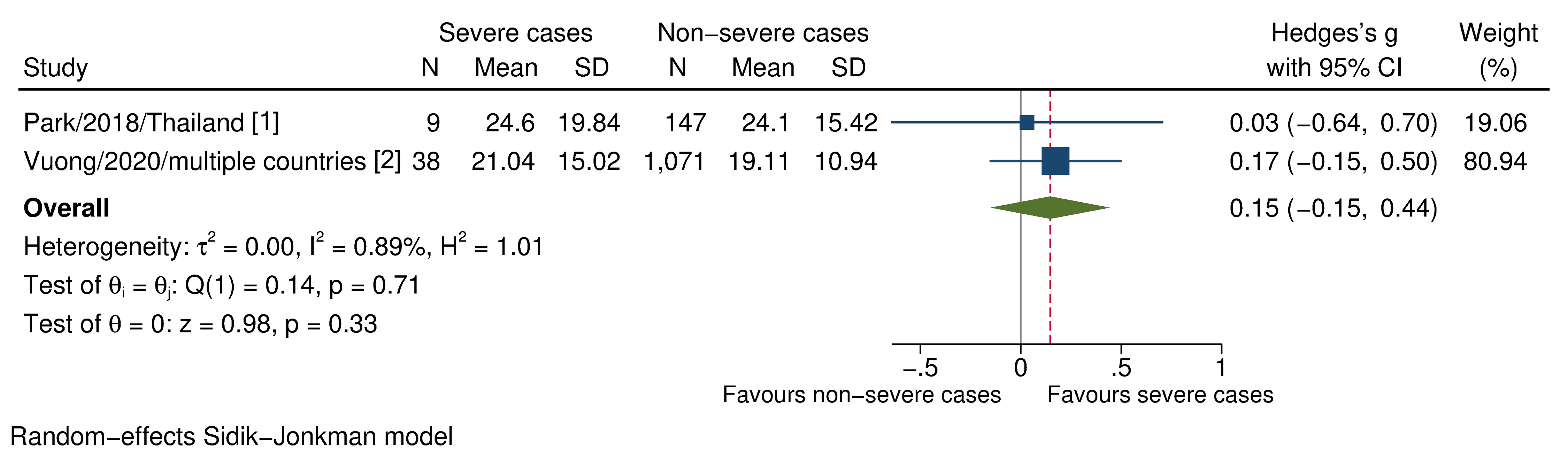

Supplement: S2 Fig — The red dashed line represented the overall effect size. (TIF) [file pntd.0009808.s002.tif]

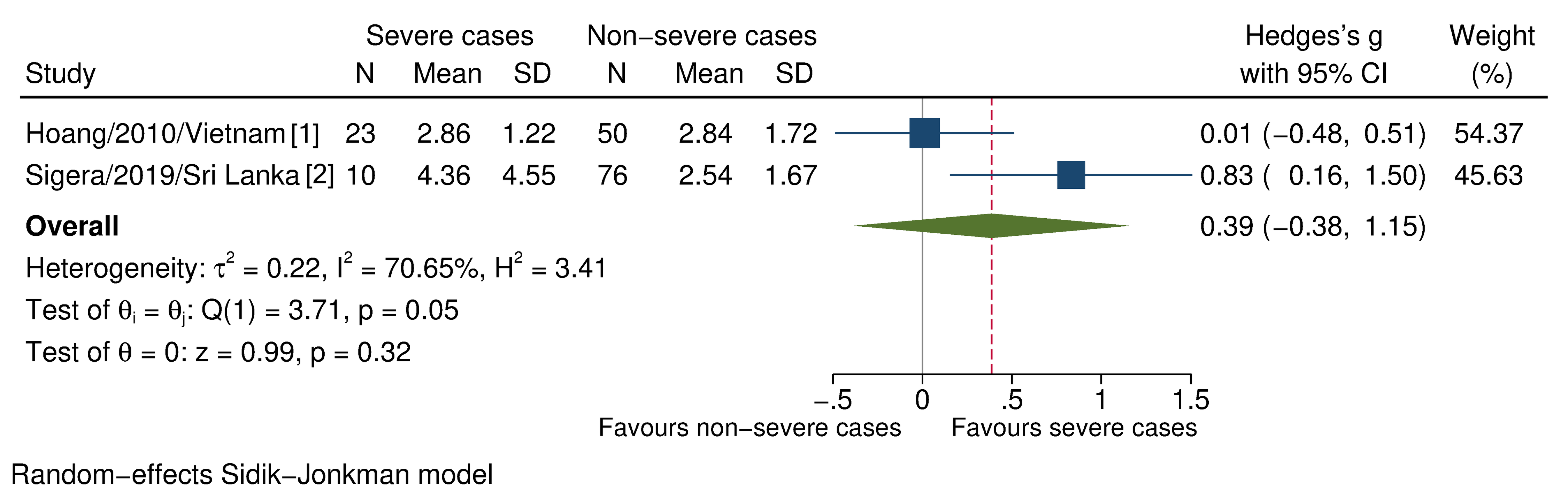

Supplement: S3 Fig — The red dashed line represented the overall effect size. (TIF) [file pntd.0009808.s003.tif]

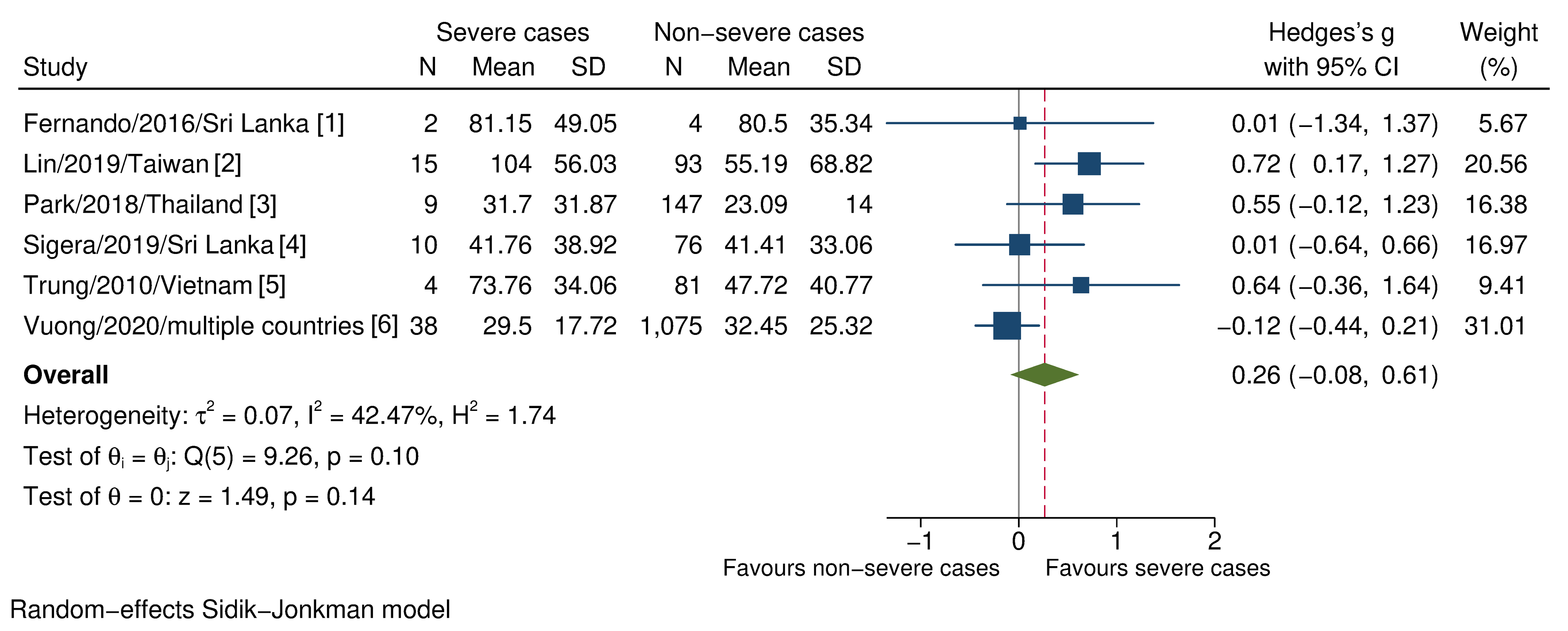

Supplement: S4 Fig — The red dashed line represented the overall effect size. (TIF) [file pntd.0009808.s004.tif]

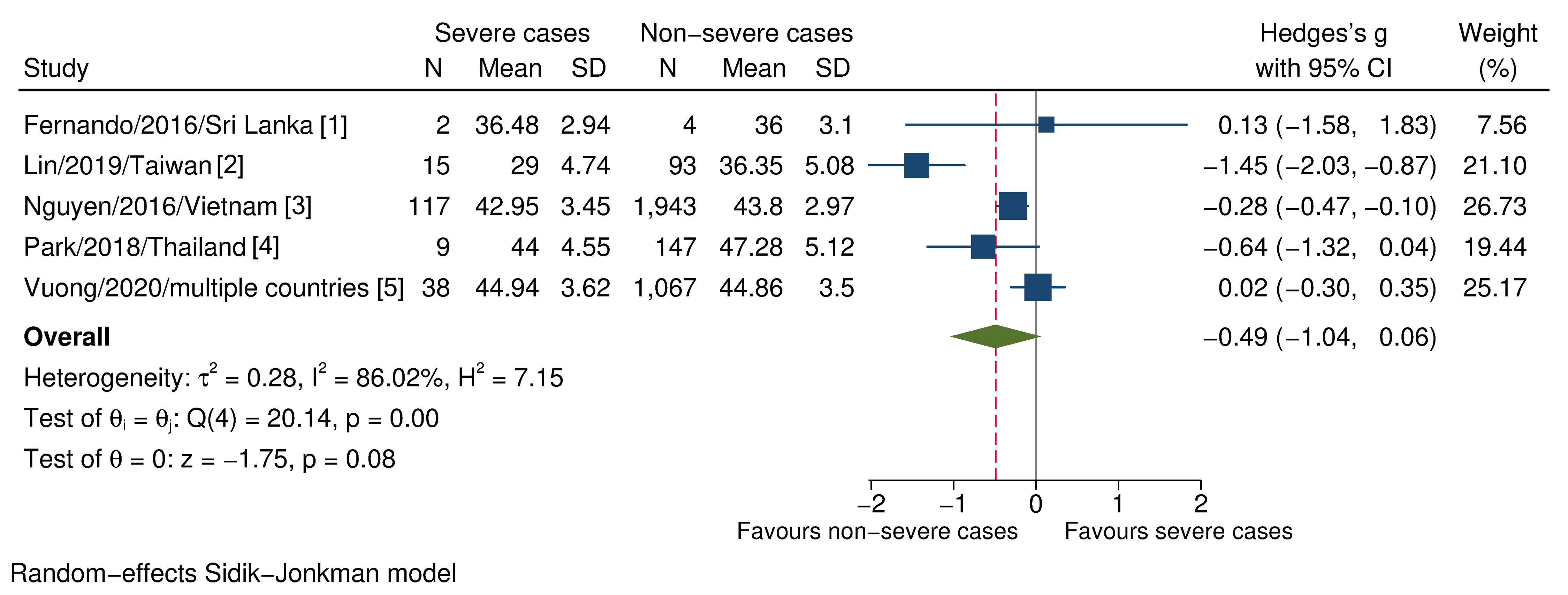

Supplement: S5 Fig — The red dashed line represented the overall effect size. (TIF) [file pntd.0009808.s005.tif]

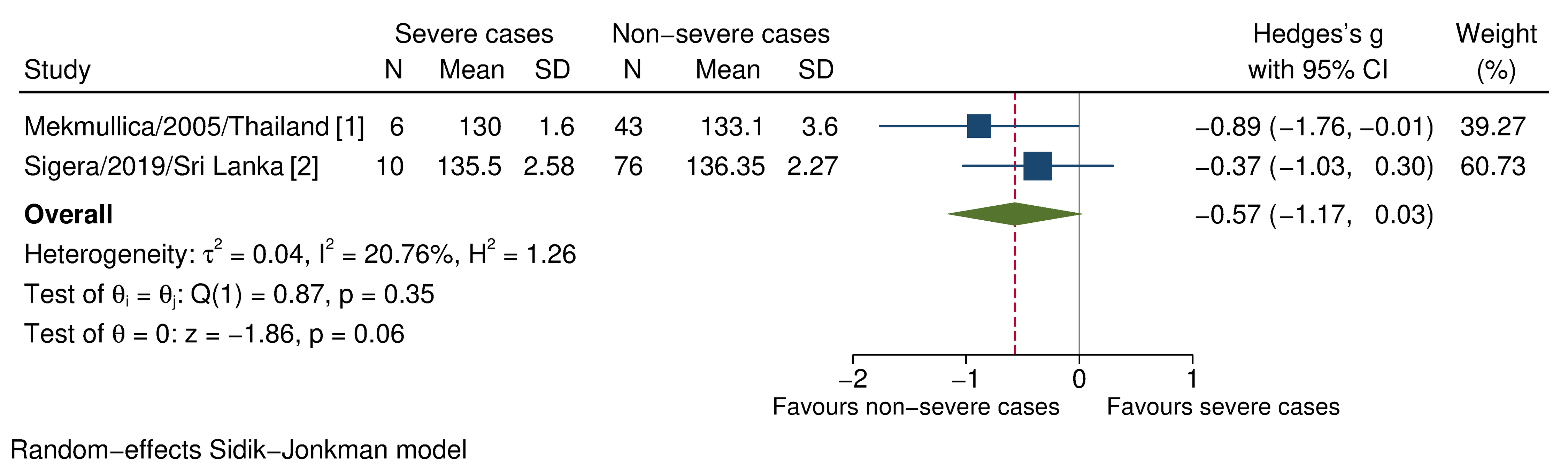

Supplement: S6 Fig — The red dashed line represented the overall effect size. (TIF) [file pntd.0009808.s006.tif]

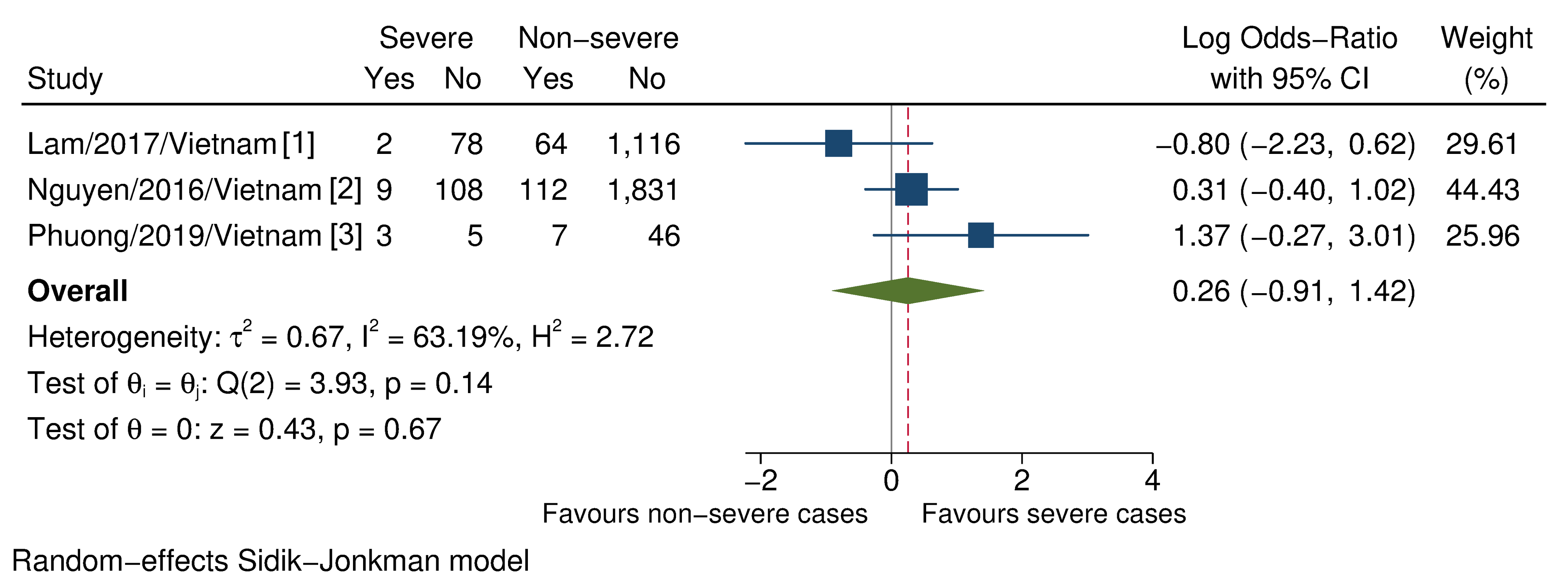

Supplement: S7 Fig — The red dashed line represented the overall effect size. (TIF) [file pntd.0009808.s007.tif]

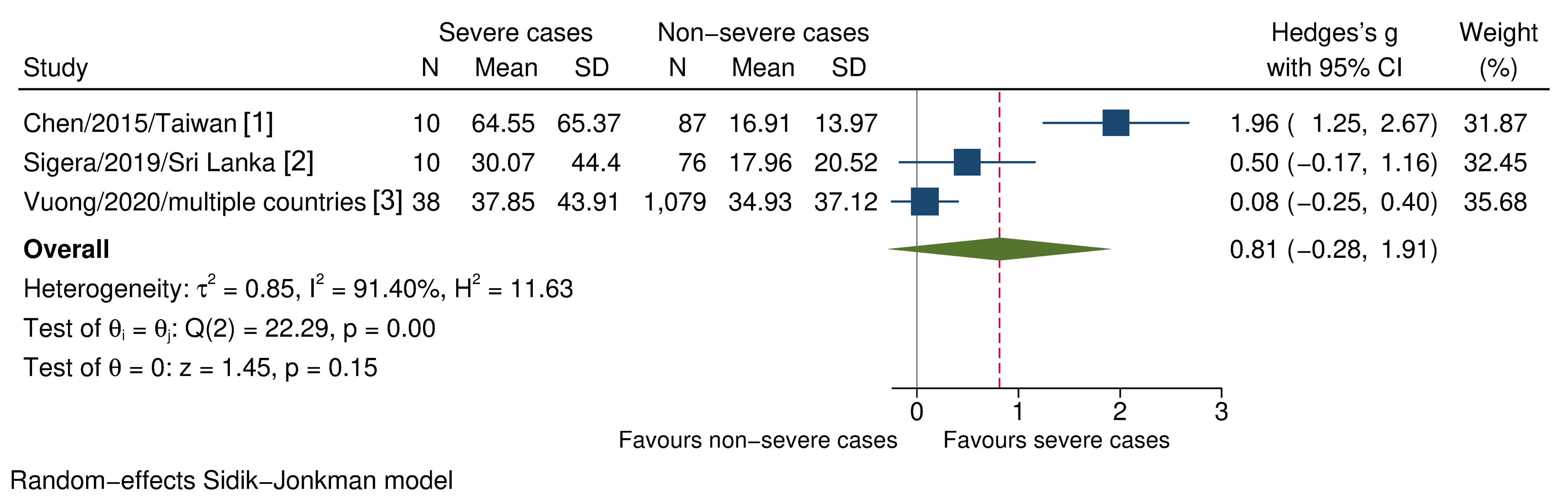

Supplement: S8 Fig — The red dashed line represented the overall effect size. (TIF) [file pntd.0009808.s008.tif]

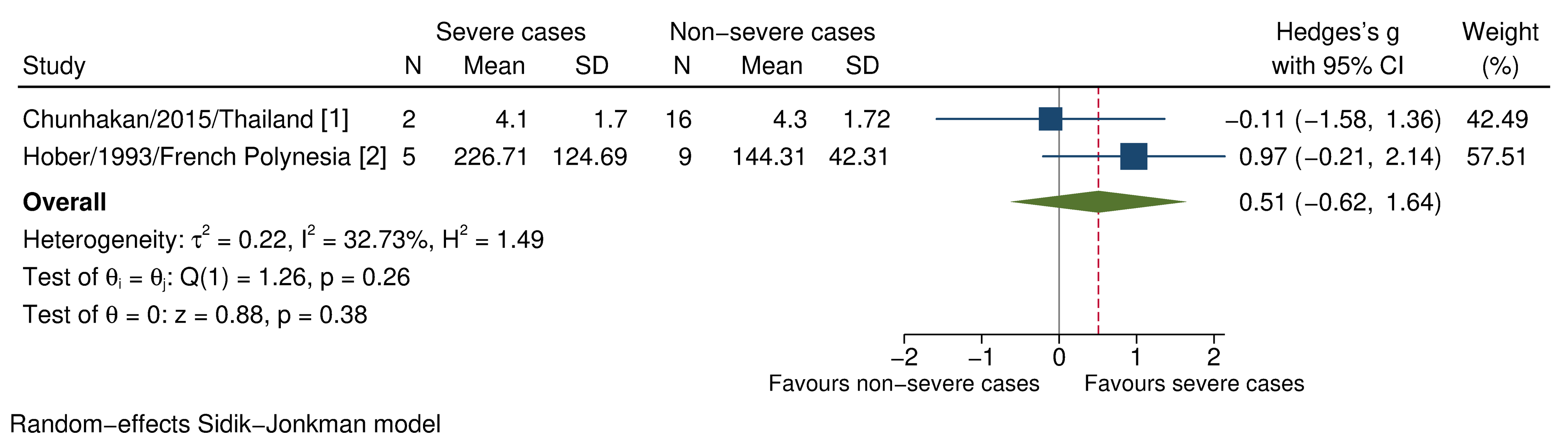

Supplement: S9 Fig — The red dashed line represented the overall effect size. (TIF) [file pntd.0009808.s009.tif]

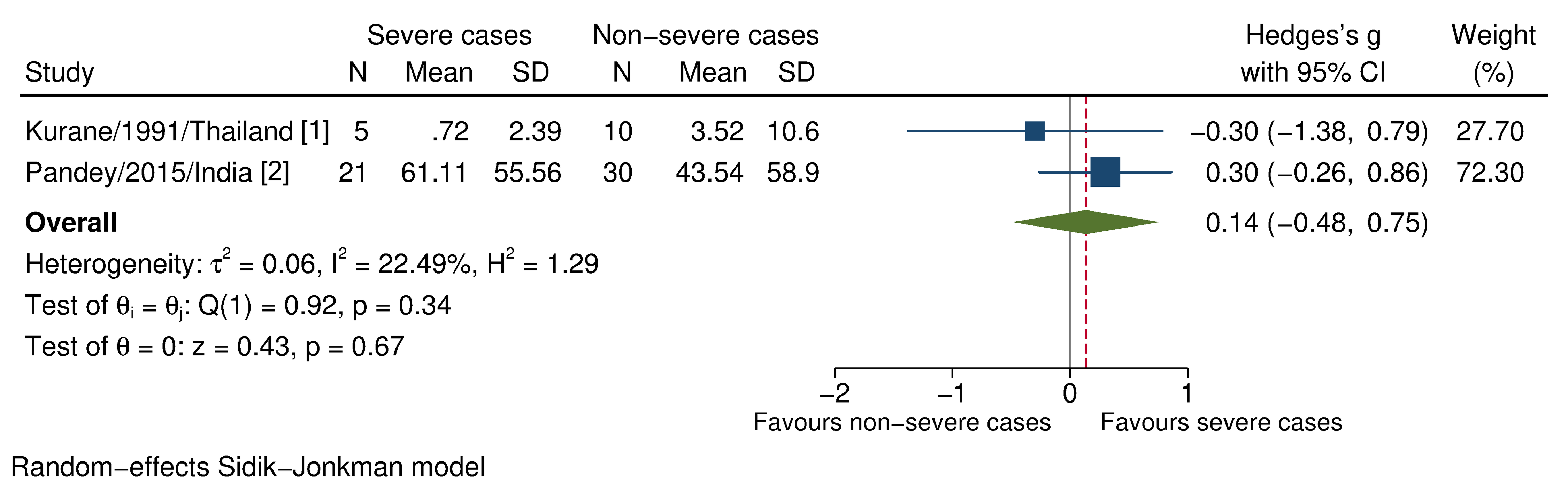

Supplement: S11 Fig — The red dashed line represented the overall effect size. (TIF) [file pntd.0009808.s011.tif]

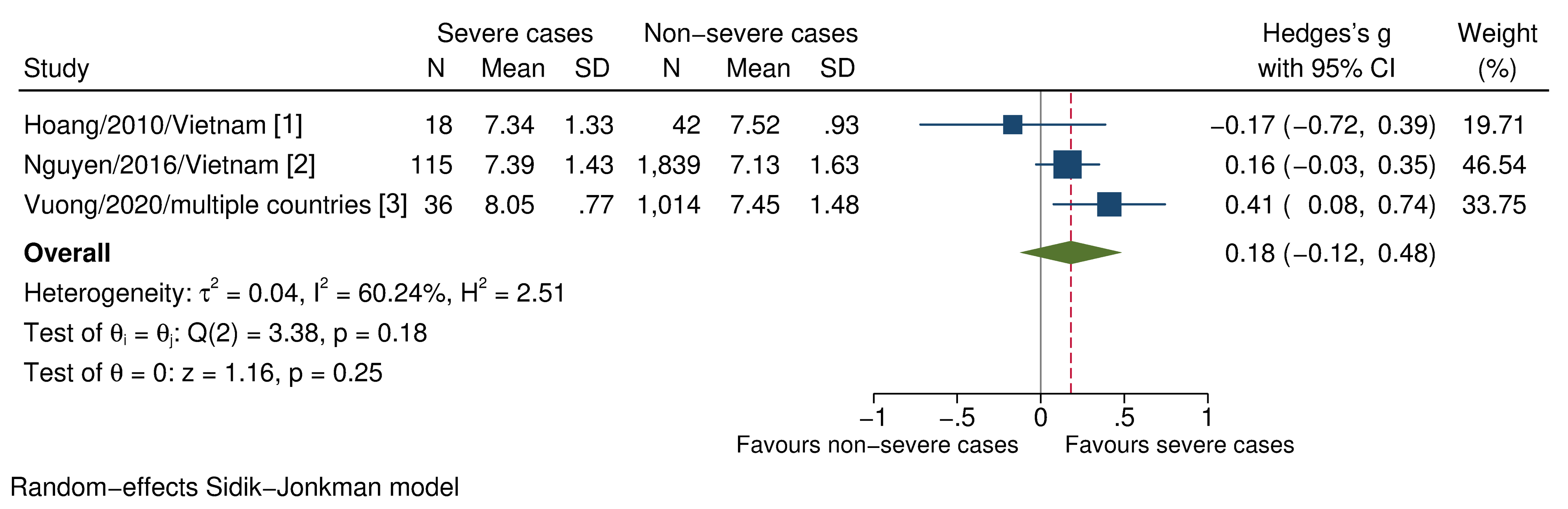

Supplement: S12 Fig — The red dashed line represented the overall effect size. (TIF) [file pntd.0009808.s012.tif]
