# Supplementary material for: Predictive markers for the early prognosis of dengue severity: A systematic review and meta-analysis
Source: PLoS Negl Trop Dis. 2021 Oct 5;15(10):e0009808. doi: 10.1371/journal.pntd.0009808 (PMC8519480; doi:10.1371/journal.pntd.0009808)
Supplement: S10 Fig — The red dashed line represented the overall effect size. (DOCX) [file pntd.0009808.s010.docx]

**S10 Fig. Forest plot showing the relationship between IL-10 levels and severe dengue.** The red dashed line represented the overall effect size.

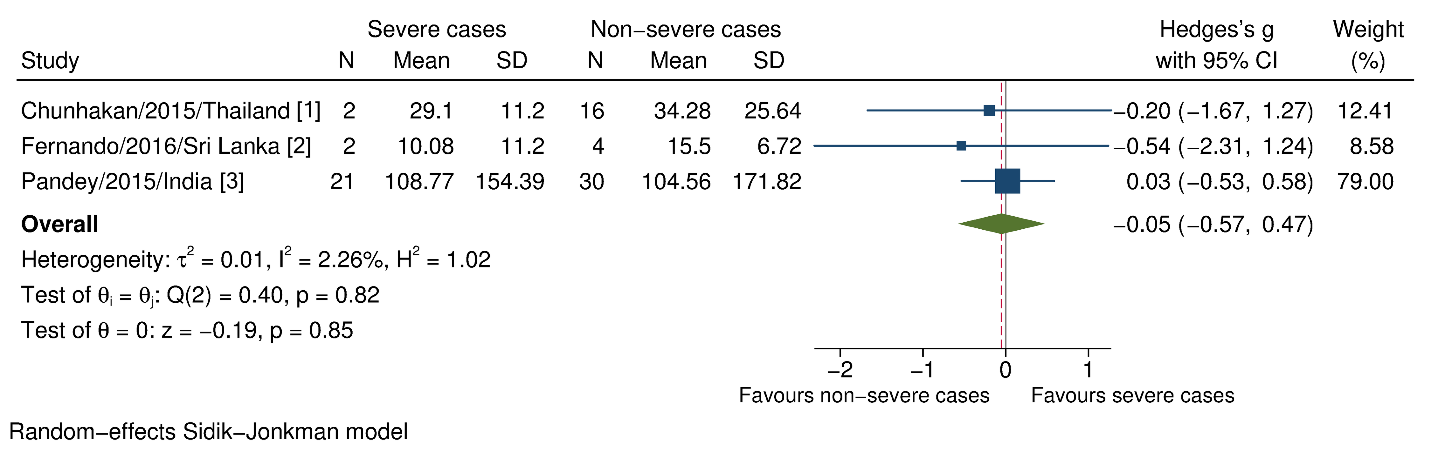


References

1. Chunhakan S, Butthep P, Yoksan S, Tangnararatchakit K, Chuansumrit A. Vascular leakage in dengue hemorrhagic Fever is associated with dengue infected monocytes, monocyte activation/exhaustion, and cytokines production. Int J Vasc Med. 2015;2015:917143.
2. Fernando S, Wijewickrama A, Gomes L, Punchihewa CT, Madusanka SD, Dissanayake H, et al. Patterns and causes of liver involvement in acute dengue infection. BMC Infect Dis. 2016;16(1):319.
3. Pandey N, Jain A, Garg RK, Kumar R, Agrawal OP, Lakshmana Rao PV. Serum levels of IL-8, IFNγ, IL-10, and TGF β and their gene expression levels in severe and non-severe cases of dengue virus infection. Arch Virol. 2015;160(6):1463-75. doi: 10.007/s00705-015-2410-6. Epub 2015 Apr 10.
