# Supplementary material for: Predictive markers for the early prognosis of dengue severity: A systematic review and meta-analysis
Source: PLoS Negl Trop Dis. 2021 Oct 5;15(10):e0009808. doi: 10.1371/journal.pntd.0009808 (PMC8519480; doi:10.1371/journal.pntd.0009808)
Supplement: S13 Fig — The estimated effects remained unchanged by excluding seven studies; the heterogeneity considerably reduced by removing an outlier [67]. (DOCX) [file pntd.0009808.s013.docx]

**S13 Fig. Sensitivity analysis showing the estimated effects of platelet counts.** The estimated effects remained unchanged by excluding seven studies; the heterogeneity considerably reduced by removing an outlier [7].

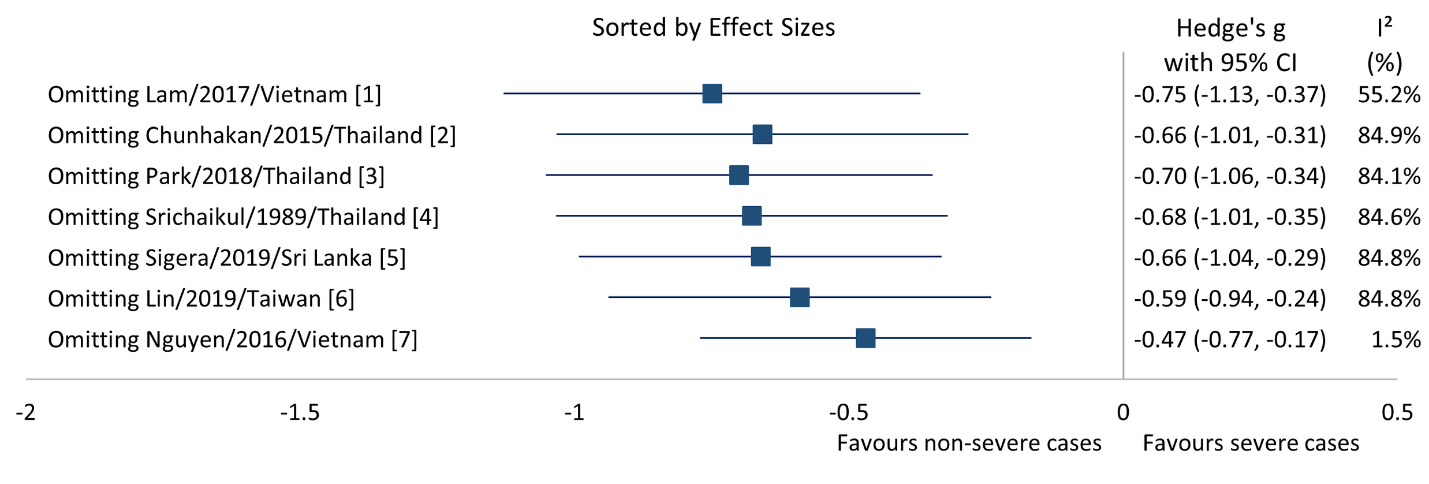

References
1. Lam PK, Ngoc TV, Thu Thuy TT, Hong Van NT, Nhu Thuy TT, Hoai Tam DT, et al. The value of daily platelet counts for predicting dengue shock syndrome: Results from a prospective observational study of 2301 Vietnamese children with dengue. PLoS Negl Trop Dis. 2017;11(4):e0005498.

2. Chunhakan S, Butthep P, Yoksan S, Tangnararatchakit K, Chuansumrit A. Vascular leakage in dengue hemorrhagic Fever is associated with dengue infected monocytes, monocyte activation/exhaustion, and cytokines production. Int J Vasc Med. 2015;2015:917143.

3. Park S, Srikiatkhachorn A, Kalayanarooj S, Macareo L, Green S, Friedman JF, et al. Use of structural equation models to predict dengue illness phenotype. PLoS Negl Trop Dis. 2018;12(10):e0006799.

4. Srichaikul T, Nimmannitya S, Sripaisarn T, Kamolsilpa M, Pulgate C. Platelet function during the acute phase of dengue hemorrhagic fever. Southeast Asian J Trop Med Public Health. 1989;20(1):19-25.

5. Sigera PC, Amarasekara R, Rodrigo C, Rajapakse S, Weeratunga P, De Silva NL, et al. Risk prediction for severe disease and better diagnostic accuracy in early dengue infection; the Colombo dengue study. BMC Infect Dis. 2019;19(1):680.

6. Lin CY, Kolliopoulos C, Huang CH, Tenhunen J, Heldin CH, Chen YH, et al. High levels of serum hyaluronan is an early predictor of dengue warning signs and perturbs vascular integrity. EBioMedicine. 2019;48:425-41.

7. Nguyen MT, Ho TN, Nguyen VV, Nguyen TH, Ha MT, Ta VT, et al. An Evidence-Based Algorithm for Early Prognosis of Severe Dengue in the Outpatient Setting. Clin Infect Dis. 2017;64(5):656-63.
