# Supplementary material for: Predictive markers for the early prognosis of dengue severity: A systematic review and meta-analysis
Source: PLoS Negl Trop Dis. 2021 Oct 5;15(10):e0009808. doi: 10.1371/journal.pntd.0009808 (PMC8519480; doi:10.1371/journal.pntd.0009808)
Supplement: S14 Fig — The estimated effects remained unchanged by excluding seven studies; the heterogeneity considerably reduced by removing an outlier [67]. (DOCX) [file pntd.0009808.s014.docx]

**S14 Fig. Sensitivity analysis showing the estimated effects of AST levels.** The estimated effects remained unchanged by excluding seven studies; the heterogeneity considerably reduced by removing an outlier [1].


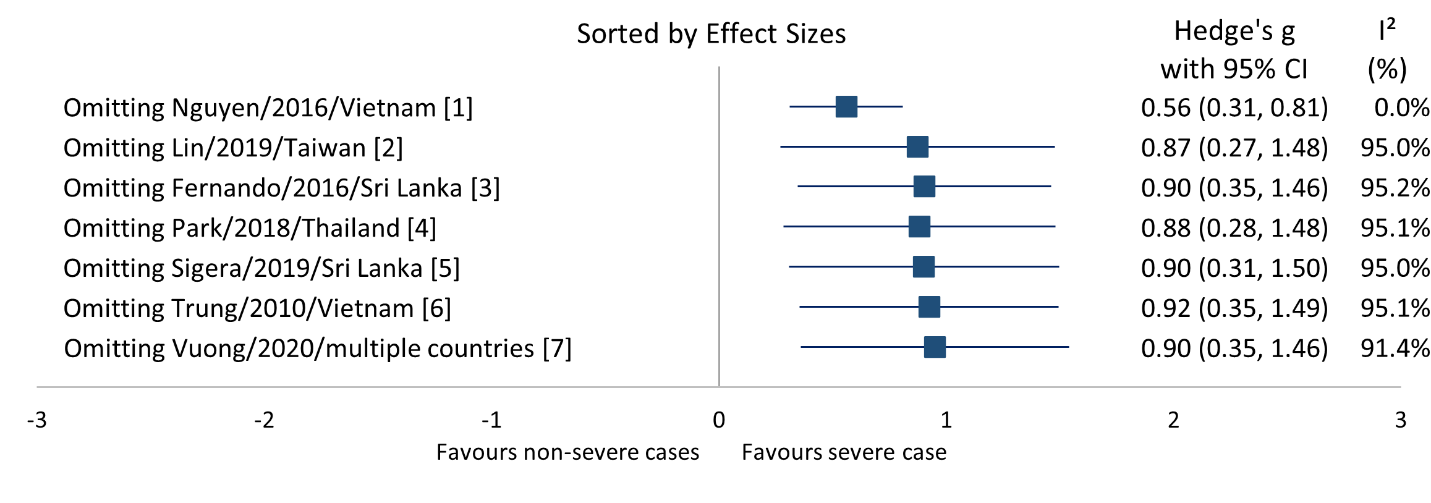


References

1. Nguyen MT, Ho TN, Nguyen VV, Nguyen TH, Ha MT, Ta VT, et al. An Evidence-Based Algorithm for Early Prognosis of Severe Dengue in the Outpatient Setting. Clin Infect Dis. 2017;64(5):656-63.

2. Lin CY, Kolliopoulos C, Huang CH, Tenhunen J, Heldin CH, Chen YH, et al. High levels of serum hyaluronan is an early predictor of dengue warning signs and perturbs vascular integrity. EBioMedicine. 2019;48:425-41.

3. Fernando S, Wijewickrama A, Gomes L, Punchihewa CT, Madusanka SD, Dissanayake H, et al. Patterns and causes of liver involvement in acute dengue infection. BMC Infect Dis. 2016;16(1):319.

4. Park S, Srikiatkhachorn A, Kalayanarooj S, Macareo L, Green S, Friedman JF, et al. Use of structural equation models to predict dengue illness phenotype. PLoS Negl Trop Dis. 2018;12(10):e0006799.

5. Sigera PC, Amarasekara R, Rodrigo C, Rajapakse S, Weeratunga P, De Silva NL, et al. Risk prediction for severe disease and better diagnostic accuracy in early dengue infection; the Colombo dengue study. BMC Infect Dis. 2019;19(1):680.

6. Trung DT, Thao le TT, Hien TT, Hung NT, Vinh NN, Hien PT, et al. Liver involvement associated with dengue infection in adults in Vietnam. Am J Trop Med Hyg. 2010;83(4):774-80.

7. Vuong NL, Le Duyen HT, Lam PK, Tam DTH, Vinh Chau NV, Van Kinh N, et al. C-reactive protein as a potential biomarker for disease progression in dengue: a multi-country observational study. BMC Med. 2020;18(1):35.
