# Supplementary material for: Predictive markers for the early prognosis of dengue severity: A systematic review and meta-analysis
Source: PLoS Negl Trop Dis. 2021 Oct 5;15(10):e0009808. doi: 10.1371/journal.pntd.0009808 (PMC8519480; doi:10.1371/journal.pntd.0009808)
Supplement: S1 Protocol — (PDF) [file pntd.0009808.s015.pdf]

Predictive markers for the prognosis of dengue severity: a systematic review and meta-analysis

Thuan Minh Tieu, Thach Tran, Manal Abdelrahman, Hazem Faraj, Ahmed Gamal Tohamy, Mario Gerges, Vincent Hou, Hang Tran, Huy Tien Nguyen

Citation

Thuan Minh Tieu, Thach Tran, Manal Abdelrahman, Hazem Faraj, Ahmed Gamal Tohamy, Mario Gerges, Vincent Hou, Hang Tran, Huy Tien Nguyen. Predictive markers for the prognosis of dengue severity: a systematic review and meta-analysis. PROSPERO 2018 CRD42018104495 Available from: http://www.crd.york.ac.uk/PROSPERO/display_record.php?ID=CRD42018104495

Review question

This study aims to generalize the characteristics of putative markers/indicators associated with the progession of dengue severity.

Searches

Relevant articles will be searched via an electronic search of Cochrane Library, Embase, POPLINE, PubMed, Scopus, SIGLE (System for Information on Grey Literature in Europe).
The search strategies will be performed for each respective database:
• Cochrane Library: “dengue (severe OR severity OR shock OR DSS OR DHF) (prediction OR predictions OR predict OR predictive OR predicted OR prognosis OR prognostic OR (logistic regression)) (early OR defervescence OR progression OR progress OR progresses OR progressed OR development OR develop OR develops OR developed)”

• Embase, POPLINE, PubMed, Scopus, SIGLE: Dengue AND (marker* OR biomarker* OR factor*) AND (sever* OR DF OR DSS OR DHF OR shock) AND (predict* OR prognos* OR correlat* OR associat* OR relat* OR (logistic regression)) AND (early OR defervescence OR progress* OR develop*)

Types of study to be included

Cross-sectional and cohort observational (prospective and retrospective) studies.

Condition or domain being studied

Dengue infection is getting more common in recent years, affecting all age groups.(1) This mosquito-borne virus disease has rapidly spread throughout the tropics with local variations, influenced by rainfall, unplanned rapid urbanization, and temperature.(2) The up-to-date-minute figures point out nearly 2.5 billion persons and 40% of the world’s population living in areas at risk of infection.(3) Approximately 50 to 100 million new dengue infections and 22,000 deaths are reported annually, predominantly in children.(4) Supportive treatment and close monitoring are now the first-line therapy given for dengue.
There are four serotypes of dengue fever virus. Recovery from one serotype does not provide complete immunity against other serotypes. A subsequent infection by the other serotypes increases the risk of developing severe dengue.(2) As usual, resolution of the febrile response occurs around days 4–7, when most patients completely recover, but other individuals can experience complications.(5) The mechanism of this progression is not yet well understood. World Health Organization (WHO) has revised the new guideline for dengue diagnosis with the amendment of warning signs. Warning signs are to aid in recognizing patients on course to develop dengue hemorrhagic fever (DHF) or dengue shock syndrome (DSS), also known as severe dengue.(6) Although the revised scheme is more sensitive to the diagnosis of severe dengue, there remain impediment to the applicability.(7) In addition, many signs are subjective and present very late.(8) The foresight of DHF and DSS are unmet. Hence, there is an urgent need to identify the predictive factors of severe dengue. The corpus of this study is to find out the reliable predictors for severe dengue in terms of clinical signs, immunological, genetic and biochemical markers.

1. Vasanwala FF, Thein TL, Leo YS, Gan VC, Hao Y, Lee LK, et al. Predictive value of proteinuria in adult dengue severity. PLoS Negl Trop Dis. 2014;8(2):e2712.
2. Qureshi AI. Ebola Virus Disease Epidemic in Light of Other Epidemics. In: Qureshi AI, editor. Ebola Virus Disease: Academic Press; 2016. p. 39-65.
3. Neiderud CJ. How urbanization affects the epidemiology of emerging infectious diseases. Infect Ecol Epidemiol. 2015;5:27060.
4. Alhaeli A, Bahkali S, Ali A, Househ MS, El-Metwally AA. The epidemiology of Dengue fever in Saudi Arabia: A systematic review. J Infect Public Health. 2016;9(2):117-24.
5. Tissera H, Rathore APS, Leong WY, Pike BL, Warkentien TE, Farouk FS, et al. Chymase Level Is a Predictive Biomarker of Dengue Hemorrhagic Fever in Pediatric and Adult Patients. J Infect Dis. 2017;216(9):1112-21.
6. Lee IK, Liu JW, Yang KD. Fatal dengue hemorrhagic fever in adults: emphasizing the evolutionary pre-fatal clinical and laboratory manifestations. PLoS Negl Trop Dis. 2012;6(2):e1532.
7. Hadinegoro SR. The revised WHO dengue case classification: does the system need to be modified? Paediatr Int Child Health. 2012;32 Suppl 1:33-8.
8. Vaughn DW, Green S, Kalayanarooj S, Innis BL, Nimmannitya S, Suntayakorn S, et al. Dengue viremia titer, antibody response pattern, and virus serotype correlate with disease severity. J Infect Dis. 2000;181(1):2-9.

Participants/population

Inclusion criteria:
• Publications reporting predictive markers in terms of clinical signs, immunology, biochemistry and gene associated with dengue severity comprising dengue fever (DF), dengue haemorrhagic fever (DHF) and dengue shock syndrome (DSS)

Exclusion criteria:
• Non-peer reviewed publications (conference, serial, theses, books, editorials, reviews, patents, comments, and correspondence)
• Studies that did not involve the infection of the whole virus (for example, vaccine research involving only recombinant proteins from virus)
• Abstract-only articles or any other studies without available full-text.
• Impossible extraction of data.
• Overlapped data sets on-human studies, if the overlapping occurs, select study with largest data.
• Animal studies and in vitro studies

Intervention(s), exposure(s)

Not applicable

Comparator(s)/control

Not applicable

Context

Primary outcome(s)

The early reliable predictive markers in terms of clinical signs, immunology, biochemistry and gene associated with dengue severity comprising dengue fever (DF), dengue haemorrhagic fever (DHF) and dengue shock syndrome (DSS).

Secondary outcome(s)

Other predictive markers considered to be a candidate to predict dengue severity.

Data extraction (selection and coding)

Data extraction will be done by two independent reviewers and the conflicts amongst the reviewers will be

consulted until the consensus is reached through the empiric counsellor. A data extraction form of the eligible articles is tabulated under Excel file by two independent reviewers so as to compile the data.

Risk of bias (quality) assessment

Two reviewers will independently assess the quality of the selected studies to evaluate the risk of bias by using Cochrane Collaboration’s tool. Each of the studies will be classified as low risk, high risk or unclear risk of bias and any conflict will be resolved with discussion till a consensus is reached. If authors are not able to achieve agreement, senior authors/supervisors will be consulted.

Strategy for data synthesis

Meta-analyses for respective factors will be performed separately using Comprehensive Meta-analysis software version 2.0 (http://www.meta-analysis.com) in which there is more than one study. The odds ratio (OR) will be computed together for both dichotomous and continuous variables when there are two groups of case and control.

The I^2 index will be used to assess heterogeneity amongst studies. Random effects and fixed effect models will be used to calculate the mean effect size of studies with significant heterogeneity (I^2 >75%) and without significant heterogeneity (I^2 <75%), respectively. Pooled OR with the corresponding 95% confidence intervals will also be calculated. We use fixed-effect model with weighting of the studies when there is a lack of significant heterogeneity (p >0.10) and use random-effects model with weighting of the studies when there is heterogeneity between studies (p < 0.10).

Analysis of subgroups or subsets

We will perform descriptive analysis and meta-analysis for subgroups if data is applicable.

Contact details for further information

Thuan Minh Tieu
tieut@mcmaster.ca

Organisational affiliation of the review

McMaster University

Review team members and their organisational affiliations

Mr Thuan Minh Tieu. McMaster University
Mr Thach Tran. Institute of Tropical Medicine (NEKKEN), Nagasaki University, Japan
Mr Manal Abdelrahman. Faculty of Medicine, Ainshams University, Egypt
Mr Hazem Faraj. Faculty of Medicine, University of Tripoli, Libya
Mr Ahmed Gamal Tohamy. Faculty of Medicine, Minufiya University, Egypt
Mr Mario Gerges. Faculty of Medicine, Alexandria University, Egypt
Mr Vincent Hou. Faculty of Health Sciences, McMaster University, Canada
Mr Hang Tran. Faculty of Medicine, Vo Truong Toan University, Vietnam
Mr Huy Tien Nguyen. Institute of Tropical Medicine (NEKKEN), Nagasaki University, Japan

Anticipated or actual start date

01 May 2018

Anticipated completion date

01 September 2018

Funding sources/sponsors

None

Conflicts of interest

Language

(there is not an English language summary)

Country
Canada

Stage of review
Review_Ongoing

Subject index terms status
Subject indexing assigned by CRD

Subject index terms
Biomarkers; Humans; Prognosis; Severe Dengue

Date of registration in PROSPERO
08 August 2018

Date of publication of this version
08 August 2018

Details of any existing review of the same topic by the same authors

Stage of review at time of this submission

Stage	Started	Completed
Preliminary searches	Yes	Yes
Piloting of the study selection process	Yes	No
Formal screening of search results against eligibility criteria	No	No
Data extraction	No	No
Risk of bias (quality) assessment	No	No
Data analysis	No	No

Versions
08 August 2018

PROSPERO
This information has been provided by the named contact for this review. CRD has accepted this information in good faith and registered the review in PROSPERO. CRD bears no responsibility or liability for the content of this registration record, any associated files or external websites.
