# Supplementary material for: Predictive markers for the early prognosis of dengue severity: A systematic review and meta-analysis
Source: PLoS Negl Trop Dis. 2021 Oct 5;15(10):e0009808. doi: 10.1371/journal.pntd.0009808 (PMC8519480; doi:10.1371/journal.pntd.0009808)
Supplement: S1 Text — (DOCX) [file pntd.0009808.s017.docx]

**Systematic search terms on nine electronic databases**

**PubMed**Dengue AND (marker* OR biomarker* OR factor*) AND (sever* OR DF OR DSS OR DHF OR shock) AND (predict* OR prognos* OR correlat* OR associat* OR relat* OR (logistic regression)) AND (early OR defervescence OR progress* OR develop*)

**Scopus**Dengue AND (marker* OR biomarker* OR factor*) AND (sever* OR DF OR DSS OR DHF OR shock) AND (predict* OR prognos* OR correlat* OR associat* OR relat* OR (logistic regression)) AND (early OR defervescence OR progress* OR develop*)

**SIGLE**Dengue AND (marker* OR biomarker* OR factor*) AND (sever* OR DF OR DSS OR DHF OR shock) AND (predict* OR prognos* OR correlat* OR associat* OR relat*OR (logistic regression)) AND (early OR defervescence OR progress* OR develop*)

**POPLINE**Dengue AND (marker* OR biomarker* OR factor*) AND (sever* OR DF OR DSS OR DHF OR shock) AND (predict* OR prognos* OR correlat* OR associat* OR relat* OR (logistic regression)) AND (early OR defervescence OR progress* OR develop*)

**COCHRANE**Dengue (severe OR severity OR shock OR DSS OR DHF) (prediction OR predictions OR predict OR predictive OR predicted OR prognosis OR prognostic OR (logistic regression)) (early OR defervescence OR progression OR progress OR progresses OR progressed OR development OR develop OR develops OR developed)

**ClinicalTrials.gov**Markers OR biomarkers OR factors OR predictors OR predict OR prognostic OR prognosis OR severe OR severity OR DF OR DHF OR DSS OR correlates OR associates OR early OR defervescence OR progress OR progression OR develop OR development | Dengue

**Embase**Dengue AND (marker* OR biomarker* OR factor*) AND (sever* OR DF OR DSS OR DHF OR shock) AND (predict* OR prognos* OR correlat* OR associat* OR relat* OR (logistic regression)) AND (early OR defervescence OR progress* OR develop*)

**GoogleScholar**dengue marker biomarker factor severe shock predict associate relate early progress develop DF OR markers OR biomarkers OR factors OR DSS OR DHF OR severity OR prognosis OR association OR relation OR defervescence OR progression OR development

**ISI**Dengue AND (marker* OR biomarker* OR factor*) AND (sever* OR DF OR DSS OR DHF OR shock) AND (predict* OR prognos* OR correlat* OR associat* OR relat* OR (logistic regression)) AND (early OR defervescence OR progress* OR develop*)

**medRxiv and bioRxiv**dengue

Note: for COCHRANE bibliographic database, the full-length search term yielded more results than the truncated one. Therefore, we used this search string as the primary term throughout the research. For POPLINE, we did not perform the update search as she was no longer available for her service since 2019.
