# Supplementary material for: Predictive markers for the early prognosis of dengue severity: A systematic review and meta-analysis
Source: PLoS Negl Trop Dis. 2021 Oct 5;15(10):e0009808. doi: 10.1371/journal.pntd.0009808 (PMC8519480; doi:10.1371/journal.pntd.0009808)
Supplement: Attachment — Submitted filename: Dengue predictors_Responses to reviewers.docx [file pntd.0009808.s020.docx]

###### Institute of Tropical Medicine

###### Nagasaki University

1-12-4 Sakamoto, Nagasaki 852-8523, Japan

Tel: +81-95-819-7818, 7820, Fax: +81-95-819-7821

E-mail: [tienhuy@nagasaki-u.ac.jp](mailto:tienhuy@nagasaki-u.ac.jp)

JUNE 19, 2021

**To Editor-in-Chief of PLOS Neglected Tropical Diseases**

**Subject:** **Revision upon manuscript**

Dear Sir,
Thank you very much for your letter regarding our manuscript—Predictive markers for the early prognosis of dengue severity: a systematic review and meta-analysis. We greatly appreciate the time and attention given upon reviewing our manuscript, and we also thank the reviewers for their comments and suggestions in detail. Our responses to each comment and suggestion have been given below.

**Responses to Reviewer 1
Method comment 1:** Overall, the methodology seemed solid, but it should be better described. It is a bit messy and not entirely clear. Statistical calculations and parameters are merely cited, which is ok. Still, it would be beneficial to count with a brief description of how they were calculated and what they represent. **Answer:** Sir, we have revised the Method systemically. For statistical calculations and parameters, we used the conventional methods and practices for conducting a meta-analysis. Therefore, we considered citing the computing methods (for Hedges’s g and LORs) instead of describing them in brief details.

In detail, we performed a meta-analysis using the Sidik-Jonkman method for a random-effect model that bears an adequate error rate in estimating the between-study variance. We used Hedges’s g—a standardized mean difference (SMD)—as the pooled estimates for continuous variables and natural logarithm odd ratios (LORs) for binary outcomes, followed by 95% confidence intervals (CIs). See page 11, line 209–215.

Furthermore, in each point estimate, methodological and clinical differences existed that might introduce noise to the inferences. Therefore, we performed subgroup analyses and meta-regression to explore these underlying differences. However, the insufficient data did not allow us to do so for all the findings. See page 11–12, line 217–225.
 **Method comment 2:** In the first place, why did the authors just use 72 hours of fever as a cutoff for febrile phase analysis? This should be specified. It is not wrong, but for adults, it could be longer, usually between 2 and 7 days. **Answer:** Sir, dengue complications usually occur on day 4–7 of the fever onset [1, 2]. Ideally, the reliable markers should have foretold the outcomes ahead of the complications’ occurrence and as soon as possible. See our explanation on page 6, line 83–84; page 7, line 102–104. This statement was more evident in Lam et al., 2017 study that provided individual patient data of 2301 participants showing the large number of patients who began to develop shock from day 4 onwards. However, only two shock cases for the first 72 hours—these two cases were determined on day 3 of fever onset [3]; this information was used for our further suggestion that was presented on page 26, line 474–477. Also, several large studies have been using this time window for their predicting, Nguyen et al., 2016; Vuong et al., 2020; and Wills et al., 2009. See Table 1. Characteristics of included studies, page 15–16 (lines were not shown in table).
The febrile stage in dengue is commonly known as lasting from 2–7 days. This refers to the possible range of fever day that one could have had. And as the febrile stage is followed by the critical phase with its complications. Therefore, we could be sure that some probably develop complications within this range. For instance, the patients with a 2-day or 3-day fever subsequently develop severe complications a few days later, say around day 4, day 5, or day 6. Taken together, we find the first 72-hour window reliable and consistent with our definition of dengue predictors. **Method comment 3:** Secondly, I still can't convince myself about grouping cases with mild dengue fever and cases with warning signs (or grade I/II of the 1997 classification) as a single non-severe group. Technically, these cases are not severe ones, ok. But they are supposed to be monitored, and sometimes they require hospitalization too. Indeed, warning signs are considered in some cases predictors of severe disease, even though this doesn't mean that their presence is mandatory for a severe outcome. So, perhaps this "warning signs" clinical category should be considered separately from the classical dengue fever and the severe cases. **Answer:** Our interest is to identify who are going to develop dengue complications and who are not. On this basis, we would have the corresponding dependent variables—with and without complications or severe dengue versus non-severe dengue, regardless of the clinical decisions that the physicians have been making.
We agree that there exist non-severe cases with the indication of observation care or hospital admission. However, these situations sometimes are careful consideration of the clinicians, especially for children. On the other hand, such decisions on observation care or hospital admission depend upon the hospitals’ guidelines, sometimes varying amongst the countries.
From the clinical perspective, the 1997 WHO classification is to identify the patients with a profound shock that the criteria for such diagnosis are to be consistent with severe plasma leak—a sort of severe dengue in the 2009 WHO classification. In other words, the combining of these two classifications is not clinically differing, but technically to say, the 2009 WHO classification has the broader scoping of severe cases [4]—severe forms of plasma leak, organ impairment, and bleeding—encompassing the severe cases identified by the 1997 WHO classification.
Also, the observational study by Mangione et al., 2014 discerned dengue shock syndrome (grade III and IV) from others in order to group it as a severe dengue form [5]. Plus, the meta-analysis study by Wang et al., 2016 combined any warning signs as classical dengue fever and defined “the criteria for DSS are the following: (1) severe plasma leakage leading to DSS; (2) fluid accumulation and respiratory distress as severe bleeding, severe organ involvement (liver, central nervous system, heart, kidneys and others)”, said the authors [6].
Despite this, we performed the subgroup analyses for platelet count and AST level and further explored this inconsistency through the GRADE approach, as shown in Table 2. GRADE evidence profile.
**Method comment 4:** Finally, even though markers were analyzed independently, according to what I assume are the meta-analysis guidelines, I wondered if there is not possible to perform a multivariate meta-analysis. Dengue physiopathological basis is multifactorial. The patient's clinical outcome depends on the balance between the genetic and immunological background of the host and viral factors, as the infecting serotype, for example. Thus, even though the data presented here is interesting, it is no "new information" when checking each factor individually. Plus, the patient's immune status (primary vs. secondary infection) that is well known and described wasn't even approached here, nor the viral factors. It is hard to believe that there are no studies available that qualify for inclusion in this analysis.  **Answer:** Thank you very much for your comment. To do so—the multivariate meta-analysis and meta-regression—the set of studies should have reported the covariates and the corresponding outcomes in the same fashion. And to maintain the statistical power in the analysis, this set should be at least ten studies [7]. Neither was met in our report—we also provided the complete data extraction showing the insufficient data reporting in the included studies. At the very primary stage to develop the protocol for this study (see Appendix 1. Protocol), these covariates and confounders were to be foreseen but impractical to integrate into the meta-analytical models as explained. And we accepted this as our major limitations, “Moreover, on the basis that dengue patients reach the clinical outcomes do so by the multifactorial interactions, on which the immune status and viral factors may have introduced noise into the findings”, see page 26, line 463–466.
**Method comment 5:** Also, I was wondering why so many studies from Latin America were excluded from your analysis. In this regard, I believe that authors should mention at least this imbalance because the host genetic background between Asian and Latin American populations might be different at some point and viral genotypes circulating in both continents. **Answer:** Indeed, they are excluded because of the unclear or late sampling time [8, 9] or mismatched classification [10]—see Appendix 5. List of excluded articles for article B431, B827, and B563 with our reasons. Still, some were included but ineligible for meta-analysis as sharing no common points with others; therefore, we principally performed a narrative review in tabular formats [11]. Only one cross-national study by Vuong et al., 2020 that included El Salvador amongst the five countries underwent meta-analysis on CRP levels [12]. Therefore, this imbalance was attributable to differing outcome of interest.
We revised as followed: “The differing time window and “severe dengue” definition resulted in a small number of eligible studies—so did the participants—included in the analyses, which impacted in the capturing of the markers and caused the population imbalances by lacking studies from Latin America and Africa—the frequent or continuous dengue risk areas—that reduced the generalizations of the markers for these sites”.
Also, our study falls within the evidence-based science that the statements about the associations between host genetic background and viral genotypes with severe dengue should be raised from our findings and observations—through narrative review or meta-analytical, but we failed to see their role done towards severe dengue by the same reason as mentioned for Method comment 3. Thus, we could not gain any evidence weight on this imbalance.
**Method comment 6—minor comments:
1.** **Lines 154-155:** Should not this be part of the search strategy?
**Answer:** Sir, these lines belonged to the Study selection section, a sub-section of the Methodology, not the Search strategy section. This may be because of our formatting. We had eight equal sub-sections in the Method section (with bigger font size than sub-sections); the search strategy, study selection, and data extraction were amongst them.
**2. Line 160:** be aware that Figure 1 is cited here for the first time, but its legend is in the results section. Please select where you consider it should appear for the first time. **Answer:** Thank you, sir. We have corrected this by removing the cited figure 1 in the Method section and keep it in the Result section. See page 12, line 234.
**3. Lines 164-167:** Should not this be part of the study selection sub-section?
**Answer:** Sir, these lines belonged to the Data extraction section, not a part Study selection sub-section.
**4. Lines 170-171:** Which criteria did the authors use for that assumption? Otherwise, please cite the corresponding reference.
**Answer:** As mentioned above, several existing pieces of evidence of the reliable time window of the 72 hours of fever onset [1, 2, 13, 14]. After this period, the patients undergo the defervescence lasting 24–48 hours with the critical complications, say usually day 4 or day 5 [1]. We have provided the corresponding references, page 9–10, line 169–170.
**5. Lines 181-184:** Please cite the corresponding references. As well, a brief description of each procedure would be helpful to understand what is being done.
**Answer:** We have revised according to your comments with references and a brief description as marked on the revised manuscript, “At the study level, we used NHLBI quality assessment and Q-genie scoring to rate the risk of bias of the component studies corresponding to their designs, case-control or cohort or genetic association studies and presented them in the characteristics of included studies table. Briefly, each study underwent a set of signalling questions about the potential bias that a study may expose to its design. We mainly based the appraisal on the resemblance of the population, including age structures, enrollment times, and locations alongside the clear sample size justification. When the reviewers could not provide sound judgement due to insufficient information, the study was of unclear risk of bias.
At the outcome level, we used the GRADE approach to grade the certainty of our findings for their clinical applicability and to generate an evidence profile, including the judgements on the risk of bias, inconsistency, imprecision, and indirectness of the findings. We graded down the robustness of evidence when serious concern arose from any of the four domains”. See page 10, line 181–194.
**6. Lines 192-193:** It is a strong claim. Be careful because many of them are not exclusive of dengue infection.
**Answer:** We thank you for this correction. We have replaced that sentence with “The markers can be biochemical substances (e.g., liver enzymes, VEGF), hematopoietic parameters (e.g., leukocyte, neutrophil), immune mediators (e.g., chemokines, cytokines), and viral footprints (e.g., viral load, NS1 antigen)—detected in any of host tissue or biological fluid, or clinical symptoms or signs, which altered or occurred due to dengue infection”. See page 11, line 202–206.

**Reviewer 1 (continued responses for result section)**
**Result comment 1:** Supplementary Information 4: Please update.
**Answer:** Sir, we apologize for this. By way of improving the table, we presented only processed data. However, raw data are to be available upon request for researchers who wish for further analyses. Please see the revised Supplementary Information 4
**Result comment 2:** It wasn't clear to this reviewer how the Kappa statistics represented the reviewers' agreement, which the authors mentioned was moderate (lines 222-224). What does this mean in terms of study inclusion? Did the authors select studies without full agreement between the three of them?
**Answer:** Sir, kappa statistic quantitates how many overlapping agreements between reviewers were made on a single choice; this method helps readers and reviewers know the quality of the screening step.
We independently screened the articles for the title/abstract screening step then submitted our choices—yes, no, and consider—to the statistician specialists, NTH and SPD, for kappa computation. The quality of this step was moderate (Κ 0.42, 95% CI 0.30 to 0.55). Next, in this step, we discussed the articles that did not get complete agreement amongst the reviewers until the interpretation satisfied. We did the same as for the full-text screening step; the quality of this step was moderate (Κ 0.56, 95% CI 0.46 to 0.65)
Besides, we set the rule that if any screening step reached the endpoint with statistically low overlapping—a low quality, we re-performed the screening. In this case, the overlapping was moderate—an acceptable quality.
And yes, we included some articles without complete agreement between the reviewers, but with discussions on the discrepancy amongst the screeners.
**Result comment 3:** It is still not completely clear to this reviewer how the calculated risk of bias was taken into account for further analysis (lines 270-272). 23 out of 40 initially selected studies presented a high risk, and some of them were included in the meta-analysis, right? Also, the "unclear" ones. Please provide a clearer explanation for this issue.

**Answer:** We had addressed this topic in the manuscript—see Table 2. Grade evidence profile for the sensitivity analyses by removing studies with the unclear or higher risk of biases. For the studies with unclear risk of biases, we have explained in detail, as shown in the revised manuscript, “When the reviewers could not provide sound judgement due to insufficient information, the study was of unclear risk of bias”. See page 10, line 188–189.

**Result comment 4:** Also, it is striking that the distinction between severe and non-severe cases wasn't available for some studies. How did the authors process that information?

**Answer:** Sir, this was because of the unmet statistical power or the small sample sizes—as is a major hindrance in dengue research as the prevalence of severe dengue is low; you can look at some large cohorts such as Nguyen et al., 2016 that recruited 1943 patients to only obtain 117 severe cases; or Lam et al., 2017 with 81 severe cases versus 1186 non-severe cases. And the studies that found no association were likely to have a small sample size; for example, Trung et al., 2010 detected no association between AST and ALT levels with severe dengue when comparing 4 severe cases versus 81 non-severe cases.
**Result comment 5:** It could be helpful maybe if studies included in the meta-analysis were somehow highlighted in Table 1.
**Answer:** We have highlighted these studies with § in Table 1.
**Result comment 6:** Lines 286-292. Maybe this information could be easily read in a table.
**Answer:** We have transformed this information into tabular formats, see page 17–18, Table 2. GRADE evidence profile.

**Result comment 7:** As mentioned in the methodology section, it concerns how results are considered without age stratification (categorization or similar). Authors claimed that "Age was homogeneous across the studies—to a certain extent" (line 296). However, this is not what is observed in Table 1. On the other side, please consider avoiding ambiguous expressions like "to a certain extent" and provide precise information instead.
**Answer:** We previously did subgroup analyses for age structure; see Table 2. GRADE evidence profile. For line 296, we indicated Phuong et al., 2019 study. However, we considered removing this line as the content would be more explicit by doing so.

**Result comment 8:** Also, regarding the severe/non-severe classification: the authors followed the same rationale as previously published meta-analysis. Still, it is questionable if results could change by considering three rather than 2 categories.
**Answer:** Our interest is to identify who are going to develop dengue complications and who are not. On this basis, we would have the corresponding dependent variables—with and without complications or severe dengue versus non-severe dengue. Therefore, considering three categories may be unnecessary; and about the results corresponding to a grouping of three instead of two, this depends on which association you seek. For example, the differences in platelet count between severe versus mild dengue—the effect could be significant, or severe versus intermediate dengue—the effect could be modest or null.
**Result comment 9:** Lines 309-310: "... instead of combining with hematocrit occurring relatively late around the critical phase" Please provide the corresponding reference.
**Answer:** We previously provided the references but in line 492 as there was no space for citing in footnotes; see page 26, line 476–477 in the revised manuscript.
**Result comment 10:** Lines 312-313: "... indicated that those with early low blood platelet counts were likely to develop severe dengue ...". In my opinion, authors should be careful about claims in terms of probabilities. This meta-analysis considers the qualitative presence/absence of a certain marker in non-severe and severe dengue cases. Though, the marker is being assessed with the clinical outcome already established. This is not a prospective study with patients being accompanied along time. Besides, even though it was discussed by the authors, platelet count and AST level are continuous variables here interpreted as qualitative, considered "low" and "elevated", respectively. What do the "low" and "elevated" represent, i.e., did the authors considered any particular cut-off? Again, these values would probably be different between adults and children, both considered indistinguishably in this analysis.
**Answer:** We thank you for this critical correction, sir. We have revised all these points according to your comments. Please refer to the revised paper for these improvements.
**Result comment 11:** Line 314: Is SMD interchangeable with Hedge's g value presented in the figures?
**Answer:** Sir, we used Hedge’s g throughout this report, and SMD represented Hedge’s g in this report. We sometimes presented SMD for its short form. We have revised upon this point for better understanding, “We used Hedges’s g—a standardized mean difference (SMD)—as the pooled estimates for continuous variables [21] and natural logarithm odd ratios (LORs) for binary outcomes [22], followed by 95% confidence intervals (CIs)”. See page 11, line 212–215.

**Result comment 12:** Figures: please include in the figures' legend the meaning of the red dashed line.
**Answer:** Thank you, we have provided this legend. “The red dashed line represented the overall effect size”.
**Result comment 13:** Lines 328-334: In line with what's mentioned above, abdominal pain, vomiting, and hepatomegaly are already considered warning signs by the WHO, which means that they are often related to severe cases. Cases presenting these signs/symptoms shouldn't, in my opinion, be considered mere "non-severe" cases. However, there is a known lack of consensus within the scientific community in this regard. Though, consider this just as an opinion and/or suggestion.
**Answer:** Sir, the 2009 WHO classification have provided such an advantageous means for healthcare workers to cope with dengue, but time is not a particular focus in this classification because researchers have observed the prevalent display of these signs during the entire course of the disease—rather than the first 72 hours of fever onset.
**Result comment 14:** Lines 353-357: It is confusing whether the outlier study from Nguyen and collaborators was or was not removed since it appeared in figures, but it is mentioned in these lines that it was removed.
**Answer:** Sir, each line in these two figures (Fig S13, S14) indicated the point estimates by omitting the corresponding study. In this case, “Omitting Nguyen/2016/Vietnam” meant that this study was removed from the meta-analysis.
**Reviewer 1 (continued responses for conclusion section)**
**Conclusion comment 1:** Lines 367-368: "successfully foretold the impending severe outcomes, with exceptionally low robustness of the evidence" Be careful with this kind of claim because the presence of abdominal pain, vomiting, and hepatomegaly are indeed warning signs that may indicate a potential progression to severe disease, but it doesn't necessarily mean that patients presenting these signs/symptoms will do so. Plus, it is also mentioned that evidence robustness was low.
**Answer:** We have revised it according to your comment. And, sir, as we explained above that, we are not really sure if these signs work in the first 72 hours. “Similarly, the presence of abdominal pain, vomiting, liver enlargement and altered hyaluronan level were suggestive of the higher risk of severe dengue progression, but with exceptionally low robustness of the evidence”. See page 21, page 344–347.
**Conclusion comment 2:** Line 369: "Clinically, bleeding is frequently present in dengue patients, irrespective of disease severity". Please provide the corresponding reference. **Answer:** We have revised and provided the corresponding references, “Thrombocytopenia is commonly seen in dengue patients, irrespective of disease severity”, see page 21, line 348–349.

**Conclusion comment 3:** Comment on grammar, especially prepositions.
**Answer:** We so much thank you for your detailed correction, and we have revised all the points you mentioned.
**Conclusion comment 4:** Line 374: "massive activation" of coagulation?
**Answer:** Massive activation of platelets, “due to the massive activation of itself, apoptosis, and bone marrow hypoplasia”. See page 21, line 353.
**Conclusion comment 5:** Lines 376-377: "Additionally, it is worth noting that the platelet decline was bound up with not only the resultant hemorrhage but also the plasma leak". Again, be careful with this kind of claim because platelet decline is typical during the febrile phase, even in patients not progressing to severe conditions. Also, it would be relative to the level of platelets you might consider as normal, low, and so on.
**Answer:** Thank you, we have removed this claim.
**Conclusion comment 6:** Lines 383-384: unclear sentence: “The raised AST alone refers to the tissue damages rather than the hepatic injury despite the fact that the interactions between sNS1 and inflammatory responses”
**Revision:** “The raised AST, in the absence of ALT elevation, reflects the tissue damages, in general, rather than the hepatic injury, even though dengue, notably circulated non-structural proteins, highly targets hepatocytes in the context of dengue tropism”, see page 21–22, line 361–364.
**Conclusion comment 7:** Lines 404-407: nor the 2009 classification consider them as severe dengue.
**Answer and revision:** We meant that vomiting and abdominal pain might be the early signs telling the imminent failure of liver and central nervous system—organ impairments, and when patients subsequently develop organ impairment, they are not classified as severe cases in the 1997 WHO classification. Therefore, the number of severe cases in the 1997 WHO classification will be smaller than that in the revised classification by lacking room for organ impairment. Moreover, the smaller sample size reduced the statistical power as well as the observed effect size.
**Revision:** “Abdominal pain, vomiting, and enlarged liver may suggest the latent impairment of organs such as the liver and central nervous system that the 1997 WHO classification did not take in as a severe form of dengue. Therefore, the effect of these signs in predicting severe outcomes would be modest in studies using such classification”, please refer to page 22, line 383–387.
**Conclusion comment 8:** Lines 427-429: unclear sentence: “Insofar, vomiting and abdominal pain fulfils their prognostic tasks, but not more of relaxing clinical management constraints against severe dengue when, for example, dengue-infected one presenting with a single vomiting episode or mild mid-abdominal pain is eligible for admission.
**Revision:** “As such, vomiting and abdominal pain fulfill their prognostic tasks, but not much to ultimately relax the clinical management pressure against dengue when using these two signs that refuse the beneficial cutoffs or hallmarks goes with the umbrella admission for observation care”. See page 23, lines 408–411.
**Conclusion comment 8:** Lines 442-444: unclear sentence: “Despite this, in themselves, our findings hold a brief for the blurred manifestation of liver damage at the early stage of dengue infection—as no evidence of ALT and albumin elevation during this period.”
**Revision:** “Despite this, the finding was consistent with our observations, supporting the unclear liver involvement at the early stage of dengue infection—as no evidence of the substantial ALT and albumin differences between the clinical severities”. See page 24, lines 424–427.
**Conclusion comment 9:** Lines 453-454: unclear sentence: “However, substantial differences allowing for the reliable prediction present mostly around the critical phase or later”
**Revision:** “However, the sonographic hallmarks allow for reliable prediction mostly around the critical phase or later”. See page 24–25, line 437–438.
**Conclusion comment 10:** Line 461: what do you mean by "late display"? The 72hs?
**Answer:** We meant the late presence of the signs, almost around the critical stage when the fever has subsided. With this late display, healthcare workers do not have enough time for their good preparedness.
**Conclusion comment 11:** Lines 474-475: What do you mean with "The evidence, needless to say, was weak and attached to the limitations of our study"?
**Answer:** Thank you, we have removed this sentence for clearer content.
**Conclusion comment 12:** Lines 479-481: unclear sentence: “For studies targeting the kinetic changes of the markers during the entire course of illness, data segregation resulted in the impaired information about the essential characteristics—as were frequently summarized for the entire period or population—and, again, lessen the sample size”
**Revision:** “Moreover, on the basis that dengue patients reach the clinical outcomes do so by the multifactorial interactions, on which the immune status and viral factors may have introduced noise into the findings. Nevertheless, these data were not always available as the authors’ interest that otherwise reported for the entire observations—longer than 72 hours of the fever onset; this existed in most of our a priori hypotheses. Therefore, we failed to collect and examine their potential impacts on the inferences thoroughly”. See page 25–26, lines 461–467.

**Reviewer 1 (continued responses for Editorial and Data Presentation Modifications)
Line 113:** DENCO abbreviation goes for?
**Answer:** We have revised upon this, “DENCO Study Group findings (DENgue Control), which were proved more sensitive to predicting severe cases”. See page 7, lines 114–115.
**Lines 120-126, 129-130, 130-131:** Please provide the corresponding references for this information.
**Answer:** For line 120–126, this was our experience while working on dengue research and learning from the empirical authors. For line 129–130 [15-18], 130–131 [19-21], we apologize for not providing the corresponding references in the main text because we did not want the authors and their collaborators found our claims offended, as the underlying reasons for doing so came from the big hindrances in dengue research—low prevalence of severe dengue. You can look at the study by Patil et al., 2018, there was only one severe case detected, which meant that the comparison was impossible. Therefore, the author would combine this single case into another group to make the comparison possible. This doing was reluctant.

**Responses to Reviewer 2
Method comment 1:** I do not have significant concerns regarding methodological aspects of this report (except that for a non-expert, these can look pretty complex). **Answer:** Sir, we have systematically revised the Method section for laypeople to keep track of what we have done. However, it sometimes requires a statistical background on systematic review and meta-analysis. We apologize for this.
**Result comment 1:** Results are clearly presented, with adequate set of figures and tables (and additional materials in appendices).
**Answer:** We so much thank you for your remarks, sir. We also have several revisions to this section. We hope that you could help us check them up.
**Conclusion comment 1:** The conclusions are consistent with the results. Limitations are described and parallels with recent literature are adequately made. **Answer:** We have also revised this part. We hope that you could help us check them up.
**Reviewer 2 (continued responses for Editorial and Data Presentation Modifications)**
**Abstract comments:** I would suggest to also mention in the abstract the markers like abdominal pain, vomiting, hepatomegaly and hyaluronan (in addition to platelets and AST, as it was done in the author summary).
**Answer:** We apologize for not doing so, as we have limited space for this part (300 words). Moreover, we have made to swap out some parts with your suggestions, but we could not be satisfactory. However, we are continually revising following your suggestion upon the abstract. **Line 99–100:** there is a confusion between DENV infection and dengue disease. The sentence could be rephrased as follows for accuracy: “DENV accounts for an annual occurrence of ~400 million infections across 129 countries, though only a ¼ is symptomatic”.
**Answer:** We have revised this part according to your comments, please refer to page 7, lines 99–100.
**Line 106:** the main issue regarding the only vaccine currently available (Dengvaxia) is not suboptimal efficacy but that this is limited to people who have been previously exposed to dengue at least once (which means necessary screening) (as this vaccine was associated with an increased risk of severe dengue in vaccinated subjects without pre-exposure).
**Revision:** “Furthermore, dengue vaccine has acquired specific achievements, but on the horns of a dilemma, restricting the vaccinations only to those with a dengue-infected history”. See page 7, lines 105–107.
**Method comments**: The Protocol (in appendix) says “empiric counsellor” and in the Methods, “empirical reviewers”. Can the role of such reviewers or counsellors be clarified?
**Answer:** Sir, we apologize for this semantic confusions. They are professors—Prof. Kenji Hirayama, Prof. Nguyen Tien Huy, and Dr. Shyam Prakash Dumre—in our department who have experience in dengue research. When our collaborators and I failed to solve the issues raised from any topic, we discussed with them to find ways out.
**Result comments**: General comment: footnotes of some tables are very long but contain important information. Might some of those information be moved to main text?
**Answer:** We have revised them according to your suggestions. Please refer to Table 1 and Table 2.
**Line 222:** is the moderate strength of agreement between the reviewers at cross-checked screening step an issue? Can it be clarified (or even discussed)?
**Answer:** It is not an issue, sir. Kappa statistic quantitates how many overlapping agreements between reviewers were made on a single choice; this method helps readers and reviewers control the quality of the screening step.
In detail, we independently screened the articles for the title/abstract screening step then submitted our choices—yes, no, and consider—to the statistician specialists, NTH and SPD, for kappa computation. The quality of this step was moderate (Κ 0.42, 95% CI 0.30 to 0.55). Next, in this step, we discussed the articles that did not get complete agreement amongst the reviewers until the interpretation satisfied. We did the same as for the full-text screening step; the quality of this step was moderate (Κ 0.56, 95% CI 0.46 to 0.65). Besides, we set the rule that if any screening step reached the endpoint with statistically low overlapping—a low quality, we re-performed the screening. In this case, the overlapping was moderate—an acceptable quality.

Sir, as usual, the kappa test is descriptive information, so they will not go further than their role.
**Comment:** Study by Nguyen et al. 2016 is found to be an "outlier" for several marker, can possible cause(s) be discussed by the authors?
**Answer:** Sir, this was because of their large sample size—117 severe dengue cases versus 1943 non-severe dengue cases compared to others. We had briefly discussed this; please refer to page 20, lines 331–335.

1.	World Health O. Dengue guidelines for diagnosis, treatment, prevention and control : new edition. Geneva: World Health Organization; 2009.

2.	Malavige GN, Ogg GS. Pathogenesis of vascular leak in dengue virus infection. Immunology. 2017;151(3):261-9.

3.	Lam PK, Ngoc TV, Thu Thuy TT, Hong Van NT, Nhu Thuy TT, Hoai Tam DT, et al. The value of daily platelet counts for predicting dengue shock syndrome: Results from a prospective observational study of 2301 Vietnamese children with dengue. PLoS Negl Trop Dis. 2017;11(4):e0005498.

4.	Katzelnick LC, Gresh L, Halloran ME, Mercado JC, Kuan G, Gordon A, et al. Antibody-dependent enhancement of severe dengue disease in humans. Science. 2017;358(6365):929-32.

5.	Mangione JN, Huy NT, Lan NT, Mbanefo EC, Ha TT, Bao LQ, et al. The association of cytokines with severe dengue in children. Trop Med Health. 2014;42(4):137-44.

6.	Wang XJ, Wei HX, Jiang SC, He C, Xu XJ, Peng HJ. Evaluation of aminotransferase abnormality in dengue patients: A meta analysis. Acta Trop. 2016;156(doi):130-6.

7.	Meta-Regression. Introduction to Meta-Analysis2009. p. 187-203.

8.	S L, L M, O B, W B, V P. Resumen. En dengue con signos de alarma ¿Podemos predecir evolución a grave desde la emergencia? Revista Chilena de Pediatría. 2015;86(4).

9.	Popper SJ, Gordon A, Liu M, Balmaseda A, Harris E, Relman DA. Temporal Dynamics of the Transcriptional Response to Dengue Virus Infection in Nicaraguan Children. PLOS Neglected Tropical Diseases. 2012;6(12):e1966.

10.	Lugo S, Pavlicich V. Indicadores tempranos de dengue grave en pacientes hospitalizados. Revista Chilena de Pediatría. 2016;87(4):326-7.

11.	Biswas HH, Gordon A, Nunez A, Perez MA, Balmaseda A, Harris E. Lower Low-Density Lipoprotein Cholesterol Levels Are Associated with Severe Dengue Outcome. PLoS Negl Trop Dis. 2015;9(9):e0003904.

12.	Vuong NL, Le Duyen HT, Lam PK, Tam DTH, Vinh Chau NV, Van Kinh N, et al. C-reactive protein as a potential biomarker for disease progression in dengue: a multi-country observational study. BMC Med. 2020;18(1):35.

13.	Yacoub S, Wills B. Predicting outcome from dengue. BMC Med. 2014;12(1):147.

14.	Lum L, Ng CJ, Khoo EM. Managing dengue fever in primary care: A practical approach. Malays Fam Physician. 2014;9(2):2-10.

15.	Salud VM, Reyes FA, Ricohermoso J, Rubion LE, Roman AD. The Reliability of C-Reactive Protein Levels in Predicting Dengue Severity: A Systematic Review. Access Microbiology. 2020;2(2).

16.	Sangkaew S, Ming D, Boonyasiri A, Honeyford K, Kalayanarooj S, Yacoub S, et al. Risk predictors of progression to severe disease during the febrile phase of dengue: a systematic review and meta-analysis. Lancet Infect Dis. 2021.

17.	Lima WG, Souza NA, Fernandes SOA, Cardoso VN, Godoi IP. Serum lipid profile as a predictor of dengue severity: A systematic review and meta-analysis. Rev Med Virol. 2019;29(5):e2056.

18.	Zhang H, Zhou YP, Peng HJ, Zhang XH, Zhou FY, Liu ZH, et al. Predictive symptoms and signs of severe dengue disease for patients with dengue fever: a meta-analysis. Biomed Res Int. 2014;2014(doi):359308.

19.	Lee YH, Leong WY, Wilder-Smith A. Markers of dengue severity: a systematic review of cytokines and chemokines. J Gen Virol. 2016;97(12):3103-19.

20.	Soo KM, Khalid B, Ching SM, Tham CL, Basir R, Chee HY. Meta-analysis of biomarkers for severe dengue infections. PeerJ. 2017;5:e3589.

21.	Htun TP, Xiong Z, Pang J. Clinical signs and symptoms associated with WHO severe dengue classification: a systematic review and meta-analysis. Emerg Microbes Infect. 2021;10(1):1116-28.

Your kind consideration will be greatly appreciated.

Sincerely,

Nguyen Tien Huy, M.D., Ph.D.

Department of Clinical Product Development,
Institute of Tropical Medicine (NEKKEN),
Nagasaki University, 1-12-4 Sakamoto, Nagasaki 852-8523, Japan
