# Supplementary material for: Predictive markers for the early prognosis of dengue severity: A systematic review and meta-analysis
Source: PLoS Negl Trop Dis. 2021 Oct 5;15(10):e0009808. doi: 10.1371/journal.pntd.0009808 (PMC8519480; doi:10.1371/journal.pntd.0009808)
Supplement: Attachment — Submitted filename: Responses to reviewers_Minor revision.docx [file pntd.0009808.s021.docx]

###### Institute of Tropical Medicine

###### Nagasaki University

1-12-4 Sakamoto, Nagasaki 852-8523, Japan

Tel: +81-95-819-7818, 7820, Fax: +81-95-819-7821

E-mail: [tienhuy@nagasaki-u.ac.jp](mailto:tienhuy@nagasaki-u.ac.jp)

SEPTEMBER 05, 2021

**To Editor-in-Chief of PLOS Neglected Tropical Diseases**

**Subject:** **Revision upon manuscript**

Dear Sir,

Thank you very much for your suggestions regarding our manuscript (PNTD-D-21-00482_R1), which have extensively helped us improve the quality of the work, and we understand that. We also thank the Referee reviewers for spending their time guiding us with detailed guidance. Our responses to each comment and suggestion have been given below.

**Responses to Reviewer 1—Editorial and Data Presentation Modification.**

Sir, we thank you for your kind guidance. For spelling errors, grammar, wrong collocations, and reference missing, we completely agreed with your suggestions and corrected those points; therefore, in this letter, we only mentioned the questions that require a full explanation. These lines are for the no-track-change source file.

**Line 113–115:** …..DENCO Study Group findings (DENgue COtrol), which were proved more sensitive to predicting severe cases [6, 7]. Looks like something is missing after “(DENgue COtrol),”. Please check and rephrase.—**Answer:** Sir, we have checked and rephrased the paragraph as follows, “In 2009, the World Health Organization (WHO) revised dengue case classification in light of multi-centre study findings known as “DENCO” (DENgue COntrol), which was proved more sensitive to predicting severe cases [6, 7]”. Please see lines 113–115.

**Line 120:** these findings. Which findings? Please try to be more specific. Also, be careful with the use of terms like “issue” because it sound vague depending on the context. For exm, in line 129. Avoid using vague terms.—**Answer:** We have corrected this sentence as follows, “Nevertheless, the reported evidence is conflicting [12-14]. These conflicts arise from insufficient study power or inconsistency in the timeframe during which markers are recorded [15, 16], often late in the disease course [15, 16].” Please see lines 120–122.

**Lines 122-124:** DHF and DSS are both severe forms of the disease, and their combination is not to compensate for low sample sizes as proposed, is because they both represent severe outcomes. Please correct this information.—**Answer:** We have corrected this sentence as follows, “Additionally, the combination of dengue hemorrhagic fever (DHF) and dengue shock syndrome (DSS) as a severe form is frequently seen [14, 17-19], which could mislead clinical triage given that management strategies differ between these two groups [20, 21].” Please see lines 122–125.

**Lines 126-127:** Not clear what the authors mean by “Given the discrepancy in methods and favored outcomes, the inconsistency in findings is inevitable”. Which methods, which favored outcomes, which findings?—**Answer:** We have removed this claim for better understanding.

**Lines 139-141:** Hard to read.—**Answer:** we have revised this sentence as follows, “We included observational studies that reported on the association of host markers and dengue severities categorized by WHO classifications.” Please see lines 137–138.

**Lines 268-269:** …. in which 57.5% of the studies were of a high risk of bias, 30.0% were of low risk of bias, and 12.5% had an unclear risk of bias. These percentages are over the 40 selected studies or the 19 included in the meta-analysis? Please clarify.

**Answer:** Sir, we meant the number of 40 studies. We revised as follows, “At the study level, risk of bias varied from low to high. Of the 40 studies, 57.5% were of a high risk of bias, 30.0% and 12.5% had low and unclear risk, respectively.” Please see lines 261–262.

**Lines 281-284:** This looks more a like a discussion. Consider moving it to the next section.—**Answer:** We have removed these lines from the legend.

**Lines 361-364:** hard to read.—**Answer:** Sir, we have revised as follows, “Moreover, AST elevation alone was more indicative of systemic inflammation than hepatic injury, although DENV highly targets hepatocytes in the context of dengue tropism [51].” Please see lines 348–350.

**Line 363:** Be careful, not all non-structural proteins. NS1 is the predominantly one, and is highly immunogenic. On the other side, it’s not that it targets hepatocytes. DENV directly infects this cell type.—**Answer:** Sir, thank you for correcting our mistake. We have revised as shown “…, although DENV highly infects hepatocytes in the context of dengue tropism [51].” Please see lines 349–350.

**Lines 371-373:** This looks inconsistent with what stated above, cited in ref 36, whose authors didn’t detect a difference in Alt o Ast levels between mild and severe dengue. Percentages are almost the same between both categories.—**Answer:** Sir, we agreed that these percentages were almost the same between severe and non-severe cases, cited in ref 36. However, it should be noted that AST and ALT, in the previous study (ref 36), were defined as >40 UI; in other words, they are binary variables. Thus, for example, the AST of 200 UI versus AST of 100 UI would make no difference when defined as >40 UI to be positive. In contrast, AST and ALT, in our study, are continuous variables that were weighed against the differences between the absolute values—mean and SD. Thus, these percentages, which were cited in our paper, described that the elevation rate of AST was higher in ALT in severe dengue, rather than comparing within them.

**Lines 378-379:** Hard to read.—**Answer:** Sir, we have revised these lines as follows, “However, our findings were unable to firmly confer their benefits in clinical practice due to weak evidence.” Please see lines 363–365.

**Lines 383-387:** For this and other reasons is that classification was updated in 2009. **Answer:** We have corrected these lines according to your suggestion, “Given that the updated WHO classification, which includes broader clinical outcomes, is more sensitive to detecting severe cases than the 1997 guideline [6, 61], the estimated effect of the markers defined was larger.” Please see lines 368–370.

**Lines 389-390:** misuse of dashes

**Lines 393-394:** Not clear at all which study uses which classification

**Lines 394-395:** 1.43 vs 1.69. Is this a relevant difference? Doesn´t look like

**Answer for these lines:** Sir, we have revised the whole paragraph as follows, “The major inconsistency in our findings regarding clinical signs was the existence of different case classifications. Given that the updated WHO classification, which includes broader clinical outcomes, is more sensitive to detecting severe cases than the 1997 guideline [6, 61], the estimated effect of the markers defined was larger. This was apparent when comparing the effects of abdominal pain between two large cohorts, Nguyen et al., 2016 versus Lam et al., 2017, corresponding to the 2009 versus 1997 WHO classification (Fig 4). In the same way, vomiting was assigned more weights in Nguyen et al., 2016 study than that by Lam et al., 2017, but as yet, the difference was weaker (Fig 5).” Please see lines 367–375.

**Line 399:** dose-response relationship? Not clear what the authors mean by that.

**Answer:** Sir, the dose-response relationship is mostly reported in intervention and epidemiology studies, referring to the modifiable outcomes with different doses or exposure levels—the higher dose, the higher response, or more exposure, higher risk. So we used this terminology as a metaphor to refer to the different clinical manifestations as dose or exposure. Please see the revised paragraph as follows, “Further, at the outcome level, bias and inconsistency may arise from the measurements of abdominal pain and vomiting. The effects could vary in terms of a “dose-response” relationship—referring to the resulting progressions of different clinical manifestations.” Line 376–379

**Lines 408-414**: Hard to read. Consider splitting into shorter sentences with more conciseness.—**Answer:** Sir, I apologize for my language. We have revised as follows, “As such, vomiting and abdominal pain fulfill their prognostic tasks, but not optimally relax case-management pressure when using these signs—which ignores the beneficial cutoffs or hallmarks—goes with the umbrella admission. It also underscores the need to properly report clinical signs, featuring how the symptoms manifest—rather than whether they do present—in association with severe dengue.” Please see lines 387–391.

**Lines 421-423:** not clear.—**Answer:** Sir, we have revised as shown, “Based on this scenario, the optimal information size is approximately 300 events in total sample size, at the power of 80% and confidence level of 95%, to capture the real effects [32]. In comparison, the number of events in our analysis was shorter than the required size to provoke a precise point estimate.” Please see lines 398–401.

**Line 452:** since Honsawek et al., 2007 to observe the raised hyaluronan level in children with DHF [76]. Please rephrase.—**Answer:** Sir, thank you. We have revised as follows, “However, few studies advance hyaluronan to explain dengue infection pathogenesis. Honsawek et al., 2007 first demonstrated the significantly increased hyaluronan level in children with DSS during the acute stage defined as days 3–7 [93].” Please see lines 428–431.

**Lines 451-466:** It can be seen that the information is there, but it is hard to read. Please rephrase, considering splitting long sentences into shorter and more concise ones.

**Answer:** We apologize for this. We have revised this paragraph. Please see lines 428–442.

**Lines 467-469:** unclear. Please rephrase.—**Answer:** Sir, we have revised as shown, “Nevertheless, such information was not always explicitly described for examining its potential impact on the inferences.” Please see lines 441–442.

**Responses to Reviewer 1—Summary and General Comments**

**Question 1)** The fact that studies' selection wasn't under full agreement of the three researchers. Instead they use a moderate Kappa estimator. This means that some sotudies wouldn't have been chosen by one of the three reviewers.
**Answer:** Sir, since we had 2666 articles for the screening step, a 100% agreement is somewhat hard to reach. For example, in a study by Bigham et al., 2011, the kappa score was between 0.65 and 0.87 [1], which signals the same scenario as in our study. We admit that the kappa score in our study was not that high compared to Bigham et al., 2011 study, but it is acceptable (moderate according to the guideline) to avoid missing data. More importantly, every difference among the three reviewers underwent the discussion to have a plausible explanation for which it made our final decision.

**Question 2)** The difficulties found to somehow merge both WHO dengue classifications (1997 and 2009) are understandable, however, as pointed out in my previous revision, it is tricky to consider cases with warning signs as mere mild dengue cases. They are not severe ones, indeed. But how sure could we be that cases published as warning signs didn't progress to severity? On the other side, the authors mentioned in the text that ws (for exm abdominal pain, vomiting, and liver enlargement) could serve as predictors for severe outcome (which is not new), but the sample size was too low. I found that contradictory in a way of saying, considering the answers given to the previous revision. Anyway, the authors made their point and were ok. I just would be very careful with conclusions obtained from this study since groups, mostly the non-severe dengue one, included very wide clinical outcomes.

**Answer:** Sir, it is reasonable when considering dengue with warning signs an important outcome, so do we. However, we also did not deem that cases with warning signs are mild dengue but non-severe dengue. Moreover, before we conducted this study, we looked over the existing research articles for their methodology to design ours [2-5]; more studies can be seen in Table 1 (in the manuscript). In other words, our method fits the clinical context. And we apologize for not giving you a satisfactory and convincing explanation from the previous letter and this.

First, as the interest of previous studies was to describe the kinetic changes between severe and non-severe dengue by time; thus, they are all longitudinal studies, following the participants from the admission (on febrile phase) to the endpoints with the final diagnosis (the severe outcomes that the patients developed).

Second, I apologize for the semantic confusion between the two manuscripts (because of my limited English). From the statistical aspect, abdominal pain, vomiting, and liver enlargement are significantly higher in severe dengue than in non-severe cases. However, from the evidence-based perspective, the evidence is of low certainty for its clinical applicability in the real world. For example, enlarged liver successfully predicts severe dengue—yes, it does—but as yet, it is not an optimum marker for use during the first 72 hours because it can yield a high false-negative prediction rate. This situation is similar to a test, considering sensitivity and specificity, in that it is relatively sensitive, say 90%, but only 67% specific to detect a disease.

Also, we know that the findings are not new in the dengue context, but the timepoint it tells the outcomes is. All the clinical signs in our paper were based on the findings of a well-designed and large study (DENCO), which associated these signs with severe dengue during the entire disease course that meant a longer 72 hours timeframe.

Your kind consideration will be greatly appreciated.

Sincerely,

Nguyen Tien Huy, M.D., Ph.D.

Department of Clinical Product Development,
Institute of Tropical Medicine (NEKKEN),
Nagasaki University, 1-12-4 Sakamoto, Nagasaki 852-8523, Japan

1.	Bigham BL, Buick JE, Brooks SC, Morrison M, Shojania KG, Morrison LJ. Patient safety in emergency medical services: a systematic review of the literature. Prehosp Emerg Care. 2012;16(1):20-35.

2.	Biswas HH, Ortega O, Gordon A, Standish K, Balmaseda A, Kuan G, et al. Early clinical features of dengue virus infection in nicaraguan children: a longitudinal analysis. PLoS Negl Trop Dis. 2012;6(3):e1562.

3.	Chen CC, Lee IK, Liu JW, Huang SY, Wang L. Utility of C-Reactive Protein Levels for Early Prediction of Dengue Severity in Adults. Biomed Res Int. 2015;2015(doi):936062.

4.	Fernando S, Wijewickrama A, Gomes L, Punchihewa CT, Madusanka SD, Dissanayake H, et al. Patterns and causes of liver involvement in acute dengue infection. BMC Infect Dis. 2016;16(1):319.

5.	Vuong NL, Manh DH, Mai NT, Phuc le H, Luong VT, Quan VD, et al. Criteria of "persistent vomiting" in the WHO 2009 warning signs for dengue case classification. Trop Med Health. 2016;44:14.
